# Editorial

**Published:** 1853-07

**Authors:** 


### THE MEDICAL EXAMINER.

PHILADELPHIA, JULY, 1853.

### PHYSICIANS FOR EMIGRANT SHIPS.

We were glad to meet with the following article in the editorial columns of the Pennsylvania Inquirer of this city. The importance of the matter commends it to all classes of the community, and we are rejoiced to see at least one of the daily papers independent enough to urge a course which, although attended with expense to shippers, must greatly advance the cause of humanity.

PHYSICIANS FOR EMIGRANT SHIPS—A GOOD SUGGESTION.—We perceive, upon looking over the Report of the Committee appointed by the American Medical Association, to memorialize Congress in favor of a law requiring every emigrant ship to have a surgeon on board, that the subject will be taken up at the coming session. It is to be hoped that the proposition will then be approved. The introduction of surgeons into all emigrant vessels, would be the means of saving many lives, while the towns and cities where emigrants are landed, would thus be largely protected against the small-pox, ship fever, and other fearful diseases. The voice of humanity is in favor of such a measure, whether we allude to the poor emigrant from abroad, or the citizen at home. It is little less than murder to fill a ship with emigrants, and thus to foment disease and promote death. The thanks of the country are due to the American Association for its energetic efforts on this interesting subject. We are all, more or less, vitally concerned in the application. The Physician on ship-board would combat the disease every inch of the way across the ocean, and by strict attention, diet, medicine and purification, would do much towards fitting the vessel to approach towards the healthy shores of the Republic. The ship fever is a dangerous disease. It requires much heroism to face, brave, and contend with it. No physician can *desire* such practice, and hence it is nothing but a conscientious sense of right and duty, that will induce a member of the medical profession to live among such patients, at sea or on land. But this sense of duty everywhere prevails, and it is an honor to the calling and to human nature. There is also another reason why such vessels as we have referred to should have surgeons. It is this: By close and constant attention to the multitude of cases that occur in crowded vessels, the physician would daily become more and more acquainted with the best mode of successfully treating ship fever, and thus of saving human life. How many medical gentlemen have already been carried off by this disease? It is, therefore, vastly important that it should be seen in all its phases, and then eventually it could and would be mastered just as certainly as our physicians now cure the intermittent fever.

The following handsome tribute to the medical profession appeared in the same paper shortly after the above, for which the author justly deserves, and should receive their warmest thanks:

PHYSICIANS—THEIR DUTIES AND THEIR LABORS.—We recently gave some extracts from the last Annual Report of the Medical Society of Pennsylvania—one of the most enlightened and benevolent bodies of the day. Our object was to show with what commendable zeal the members of the Medical Profession were laboring in the cause of humanity. All their efforts tend to the health and happiness of our race. What a vast volume of knowledge do they open up, for the examination and consideration of the intellectual and gifted in all parts of the world!

The Doctor is not only a prescriber for the sick or wounded, but he is a Botanist, a Chemist, a Geologist and Mineralogist—aye, everything. There is no *leaf* in any book of science, that he does not carefully study, with the view of bringing to the bedside of his patients something that can aid him in curing disease.

The Medical Profession is emphatically the *learned* profession. Nor do its members become weary in well doing. Every year they assemble in some of our large cities, to compare notes with one another, and to discuss what has been discovered within the preceding year. What able Reports are there made and distributed throughout the nation!

The annual publications of the American Medical Convention teem with profound intelligence upon the improvement and advances in their great art. Nor do they ask any pecuniary assistance from the other classes of society to meet their expenses in journeying to or remaining at their intellectual gatherings.

These are not the ordinary conventions of the day. They meet for the noblest and best of purposes—to smooth the pathway of man through this stormy wilderness of life. Like their great Master, who went abroad healing the sick and cleansing the lepers, they are endeavoring to walk in his benevolent footsteps. And when the last summons must come, how often are they the only persons capable of giving consolation to the dying, as the soul takes its departure from its clay tenement, and wings its way to immortality!

What vast interests are committed to their hands! and we think we may say right justly—for we know of no body of men who peril more in the cause of humanity. Pestilence cannot scare them, nor does squalid poverty pass neglected by them. We have all seen them take their lives in their hands, in performing the high duties committed to their trust. We may well say, therefore, that we most sincerely thank the members of that noble profession for their constant study to advance the science they so eminently honor. Their Conventions are, in truth and in fact, Conventions *for* us, for the elevation of suffering humanity, for we all belong to that vast brotherhood.

---

### DR. E. BROWN-SEQUARD.

This distinguished physiologist leaves the United States, to return to Paris, on the 30th of July. We are desired to announce that he will take charge of and transmit any books, pamphlets or communications for the "*Societé de Biologie*" of Paris. His address is No. 17 University Place, New York.

He will carry with him the best wishes of the many friends whom he has made during his residence in this country.

